# Ameliorative Effects of Exogenous Proline on Photosynthetic Attributes, Nutrients Uptake, and Oxidative Stresses under Cadmium in Pigeon Pea (*Cajanus cajan* L.)

**DOI:** 10.3390/plants10040796

**Published:** 2021-04-19

**Authors:** Khizar Hayat, Jafar Khan, Asif Khan, Shakir Ullah, Shahid Ali, Salahuddin, Yujie Fu

**Affiliations:** 1 Key Laboratory of Plant Ecology, Northeast Forestry University, Harbin 150040, China; khizarhayat637nefu@gmail.com (K.H.); qw4133881@nefu.edu.cn (J.K.); shakirshamas321@gmail.com (S.U.); 2 Department of Botany, Abdul Wali Khan University, Mardan 23200, Pakistan; asif.khan@usp.br; 3 College of Life Sciences, Northeast Forestry University, Harbin 150040, China; Shahidali@nefu.edu.cn; 4 Agriculture Research Station, Harichand 24520, Pakistan; salaaup@gmail.com

**Keywords:** antioxidant enzymes, *Cajanus cajan*, exogenous proline, metal stress, growth traits

## Abstract

Proline plays a significant role in the plant response to stress conditions. However, its role in alleviating metal-induced stresses remains elusive. We conducted an experiment to evaluate the ameliorative role of exogenous proline on cadmium-induced inhibitory effects in pigeon pea subjected to different Cd treatments (4 and 8 mg/mL). Cadmium treatments reduced photosynthetic attributes, decreased chlorophyll contents, disturbed nutrient uptake, and affected growth traits. The elevated activity of antioxidant enzymes (superoxide dismutase, catalase, and glutathione peroxidase), in association with relatively high contents of hydrogen peroxide, thiobarbituric acid reactive substances, electrolyte leakage, and endogenous proline, was measured. Exogenous proline application (3 and 6 mM) alleviated cadmium-induced oxidative damage. Exogenous proline increased antioxidant enzyme activities and improved photosynthetic attributes, nutrient uptake (Mg^2+^, Ca^2+^, K^+^), and growth parameters in cadmium-stressed pigeon pea plants. Our results reveal that proline supplementation can comprehensively alleviate the harmful effects of cadmium on pigeon pea plants.

## 1. Introduction

Anthropogenic sources have been continuously adding heavy metals to the soil [1,2]. This contamination inflicts detrimental effects on ecosystems, affecting their biotic components, including animals and plants [3,4]. Plants, due to being sessile and directly dependent on the soil, frequently experience the harmful effects of these heavy metals [5]. Among the heavy metals, cadmium is a highly toxic, non-degradable pollutant that significantly impacts living organisms [6,7,8]. It is discharged into the environment from metalworking industries, power stations, heating systems, and urban traffic [2,9]. Despite its exclusion from the essential nutrient category, plants eagerly absorb and accumulate cadmium (Cd) in their respective tissues [6,10]. Its existence in agricultural soil poses a severe threat due to its entry into the food chain, thus damaging animal and human health [11,12,13]. Elevated cadmium quantities in plants delay seed germination, induce growth inhibition, and reduce productivity [14,15,16]. It plays an influential role in the uptake, transport, and distribution of nutrients [17,18,19,20]. Its destructive role in the photosynthetic apparatus has been highlighted, where it reduces pigment contents [21]. Cadmium decreases carbon assimilation, reshaping the chloroplast ultra-structure and thylakoid composition [22,23,24]. Its strong affinity toward sulfate and the portentous component of enzymes leads to enzyme inhibition [25]. Cd is non-redox active, particularly interrupting homeostatic cellular redox, resulting in the excessive production of reactive oxygen species (ROS) [24,26,27].

Plant metabolic activities generate ROS in small amounts. Under stress conditions, this ROS generation exceeds the maximum level [24,27], leading to oxidative stresses [28,29]. The consequences of oxidative stresses in plants involve lipid peroxidation, electrolyte leakage, and damage to membranes and DNA molecules [24,30,31]. Nature has gifted plants with physiological and chemical defenses that are triggered by stress conditions. The physiological and chemical defenses of plants consist of non-enzymatic and enzymatic antioxidant components [32,33]. Non-enzymatic defense mechanisms include phenolics, proline, ascorbic acid, glutathione, and many other stress molecules. Superoxide dismutase, peroxide reductase catalase, ascorbate peroxidase, glutathione peroxidase, etc., constitute the enzymatic defense. These different antioxidants co-ordinate in an organized mechanism to protect delicate cellular entities, such as membranes, lipids, proteins, and nucleic acids, from oxidative stress injuries [34,35,36,37].

Proline is an organic osmoprotectant that accumulates in comparative amounts in plants under abiotic stresses [38,39]. It performs multiple functions in plants, including stress tolerance, signaling, radical scavenging, and protein stabilizing, and serves as a nutrient reservoir [39,40,41]. Its exogenous application is also effective in the alleviation of abiotic stresses. Several manuals have highlighted its ameliorative role in various stress environments [42,43,44,45,46]. Shahid et al. reported that exogenous proline improved antioxidant enzymes and the water status of pea plants under nickel stress [47]. Similarly, Zouari et al. reported the enhanced growth traits, photosynthetic activities, and antioxidant defense of olive plants in a cadmium stress medium [48].

Pigeon pea (*Cajanus cajan* L.) is a member of the family Fabaceae. It is a fast-growing shrub that is cultivated in the tropic and semi-tropic regions of the world [49,50]. Its seeds are a rich source of proteins. Its green pods are used as vegetables, whereas the husks and leaves are used as fodder for livestock. It is also used as a medicinal plant [49,50]. In traditional Chinese medicine, it is used as a sedative to relieve pain; its young leaves are boiled to kill worms, arrest the blood, and enhance the immune system [51]. To the best of our knowledge, no previous work has been carried out on the impact of soil cadmium on pigeon pea. Therefore, the purpose of this present research was to: (1) evaluate the response of pigeon pea in a cadmium stress medium and (2) investigate whether exogenous proline can alleviate cadmium-induced toxicity in pigeon pea, and, if so, to determine the possible mechanism through which this proline-mediated protection occurs. Thus, plant growth, photosynthetic pigments, gas exchange, oxidative damage, antioxidant enzymes, and tissue proline under cadmium stress with or without exogenous proline were assessed.

## 2. Results

### 2.1. Cadmium Accumulation

The cadmium accumulation in pigeon pea tissues (roots and leaves) for different cadmium treatments, both alone and in combination with exogenous proline, is shown in Table 1. Cadmium mostly accumulated in the roots, whereas a small quantity translocated to the leaves. This higher cadmium accumulation in the roots was observed for both cadmium treatments. In plants treated with 8 mg/mL Cd, amounts of 329.3 and 161.26 μg/g of cadmium were measured in the roots and leaves. Exogenous proline in the cadmium irrigation solution reduced cadmium accumulation in pigeon pea, where its effect on cadmium reduction was dose-dependent (*p* ≤ 0.05, Table 1). The addition of 6 mM exogenous proline in 8 mg/mL Cd irrigation solution reduced cadmium by 151.5% in leaves and 267.5% in roots in comparison with the 8 mg/mL Cd treatment alone.

### 2.2. Nutrient Uptake

The effect of cadmium treatments, both alone and in combination with exogenous proline, on nutrient uptake (i.e., Mg^2+^, Ca^2+^, and K^+^) is described in Table 1. The macronutrient uptake response was different under the various cadmium treatments. Under low cadmium stress, Mg^2+^ uptake increased, Ca^2+^ decreased, and K^+^ remained unchanged in leaves. In contrast, under high cadmium stress, the uptake of these nutrients reduced in leaves and roots compared with control plants (Table 1). Exogenous proline in cadmium irrigation solution enhanced the nutrient uptake of K^+^, Ca^2+^, and Mg^2+^. The addition of 6 mM exogenous proline in 8 mg/mL cadmium irrigation solution increased K^+^, Ca^2+^, and Mg^2+^ by 22.08%, 33.36%, and 14.11% in leaves and 21.78%, 29.11%, and 11.05% in roots, respectively, compared with 8 mg/mL Cd treatment alone.

### 2.3. Chlorophyll Content

Different cadmium stress effects, both alone and in combination with exogenous proline, on chlorophyll contents are shown in Table 2 (*p* ≤ 0.05). The obtained results show a significant reduction in chlorophyll content at both cadmium stress levels compared with control plants. Exogenous proline in the cadmium irrigation solution enhanced chlorophyll contents (*p* ≤ 0.05, Table 2). The addition of 6 mM exogenous proline in 8 mg/mL cadmium irrigation solution increased chlorophyll a and chlorophyll b by 67% and 49%, respectively, compared with Cd 2 treatment alone.

### 2.4. Gas Exchange Attributes

The impact of cadmium alone and in combination with exogenous proline on gaseous exchange attributes, that is, net photosynthesis (Pn), transpiration rate (E), and stomatal conductance (Gs), is presented in Table 2. The results indicate a remarkable decline in gaseous exchange attributes under cadmium exposure compared with control plants (*p* ≤ 0.05, Table 2). Proline addition in the cadmium irrigation solution improved net photosynthesis, transpiration rate, and stomatal conductance in pigeon pea. In comparison with 8 mg/mL cadmium treatment alone, 6 mM exogenous proline in 8 mg/mL cadmium irrigation solution improved net photosynthesis by 43%, stomatal conductance by 37%, and transpiration rate by 29%.

### 2.5. Growth Parameters

The effects of cadmium treatments, alone and in combination with exogenous proline, on plant height, leaf area, and dry masses (leaves and roots) are shown in Table 3. Cadmium stress reduced height, leaf area, and dry masses (leaves and roots) compared with control plants. Proline addition in the cadmium irrigation solution reduced the adverse effects of cadmium on growth parameters. In comparison with the value calculated for the 8 mg/mL cadmium treatment alone, 6 mM exogenous proline addition in the 8 mg/mL cadmium irrigation solution improved plant height by 11%, leaf area by 29%, leaf dry mass by 14%, and root dry mass by 16%, as shown in Table 3.

### 2.6. Oxidative Stress Indicators

**Figure 1:**
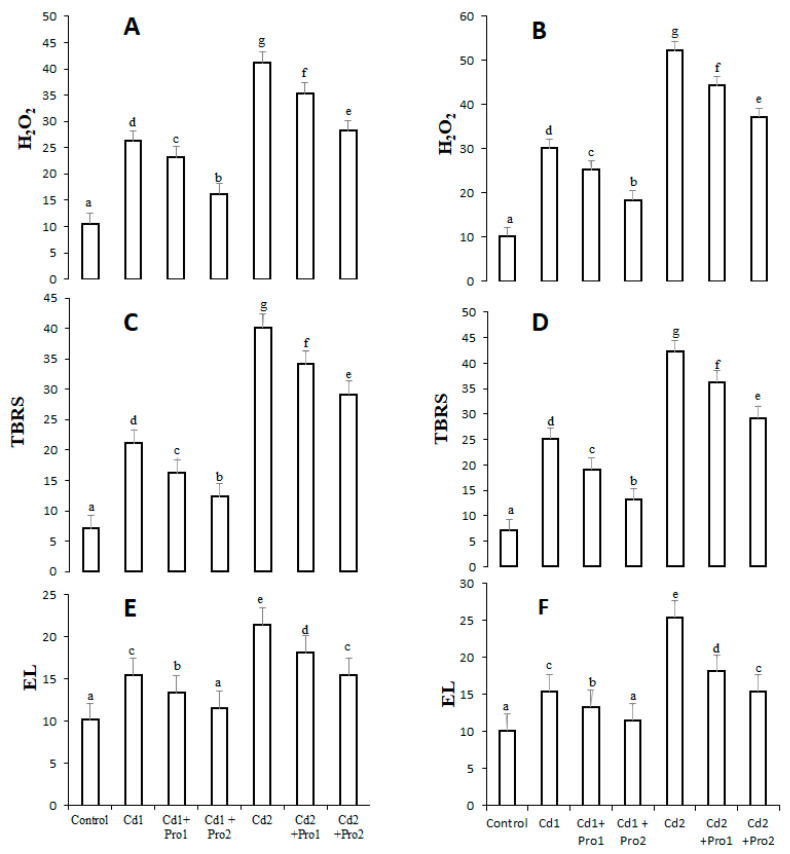
Hydrogen peroxide (H_2_O_2_, mmol g^−1^ FW) and lipid peroxidation (TBRS) contents (mmol g^−1^ FW), and electrolyte leakage (EL%) in the leaves (**A**,**C**,**E**) and the roots (**B**,**D**,**F**) of pigeon pea exposed to different Cd and exogenous proline treatments. Bars represent the mean of three replicates ± SD. Cd 1 (4 mg), Cd 2 (8 mg), Cd 1 + Pro 1 (4 mg Cd + 3 mM proline), Cd 1 + Pro 2 (4 mg Cd + 6 mM proline), Cd 2 + Pro 1 (8 mg Cd + 3 mM proline), Cd 2 + Pro 2 (8 mg Cd + 6 mM proline). Different small letters show significant differences, and the same small letters show non-significant differences according to Duncan’s multiple range test (*p* ≤ 0.05).

The effects of cadmium stress alone and with the addition of proline on hydrogen peroxide and lipid peroxidation (TBRS) contents as well as electrolyte leakage (EL) are illustrated in Figure 1. The results indicate a significant increase in oxidative stress indicators under both cadmium treatments. In 8 mg cadmium-stressed plants, H_2_O_2_, TBRS, and EL contents increased by 298%, 294%, and 96% in leaves and 385%, 321%, and 111% in roots, respectively, compared with control plants (Figure 1). Interestingly, exogenous proline decreased the oxidative stress indicators in cadmium-treated plants. In contrast with the 8 mg/mL Cd treatment alone, 6 mM exogenous proline addition to the 8 mg/mL cadmium irrigation solution decreased H_2_O_2_, TBRS, and EL contents by 121%, 105%, and 46% in the leaves and 147%, 121%, and 58% in the roots, respectively, of pigeon pea.

### 2.7. Effect on Endogenous Proline

**Figure 2:**
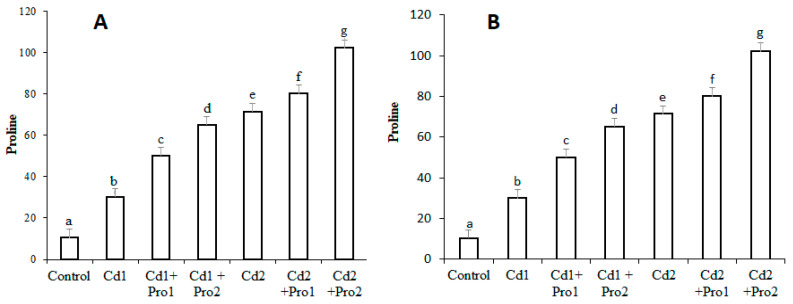
Proline contents (mmol g^−1^ fresh weight (FW)) (**A**) in the leaves and (**B**) in the roots of pigeon pea plants, under different cadmium and exogenous proline treatments. Bars represent the mean of three replicates ± SD. Cd 1 (4 mg), Cd 2 (8 mg), Cd 1 + Pro 1 (4 mg Cd + 3 mM proline), Cd 1 + Pro 2 (4 mg Cd + 6 mM proline), Cd 2 + Pro 1 (8 mg Cd + 3 mM proline), Cd 2 + Pro 2 (8 mg Cd + 6 mM proline). Different small letters show significant differences (*p* ≤ 0.05) according to Duncan’s multiple range test.

The effects of cadmium stress, alone and in combination with exogenous proline, on endogenous proline are shown in Figure 2. Endogenous proline increased in the roots and leaf tissue of pigeon pea under both cadmium treatments (*p* ≤ 0.05; Figure 2). The maximum increases (63% and 51%, respectively) were measured in the roots and leaves of Cd 2-treated plants compared with the control. Exogenous proline addition to the cadmium irrigation solution further enhanced the endogenous proline content. In combined exogenous proline and cadmium treatment, 91% and 82% increases in endogenous proline were observed in roots and leaves, respectively (Figure 2).

### 2.8. Antioxidant Enzyme Activities

**Figure 3:**
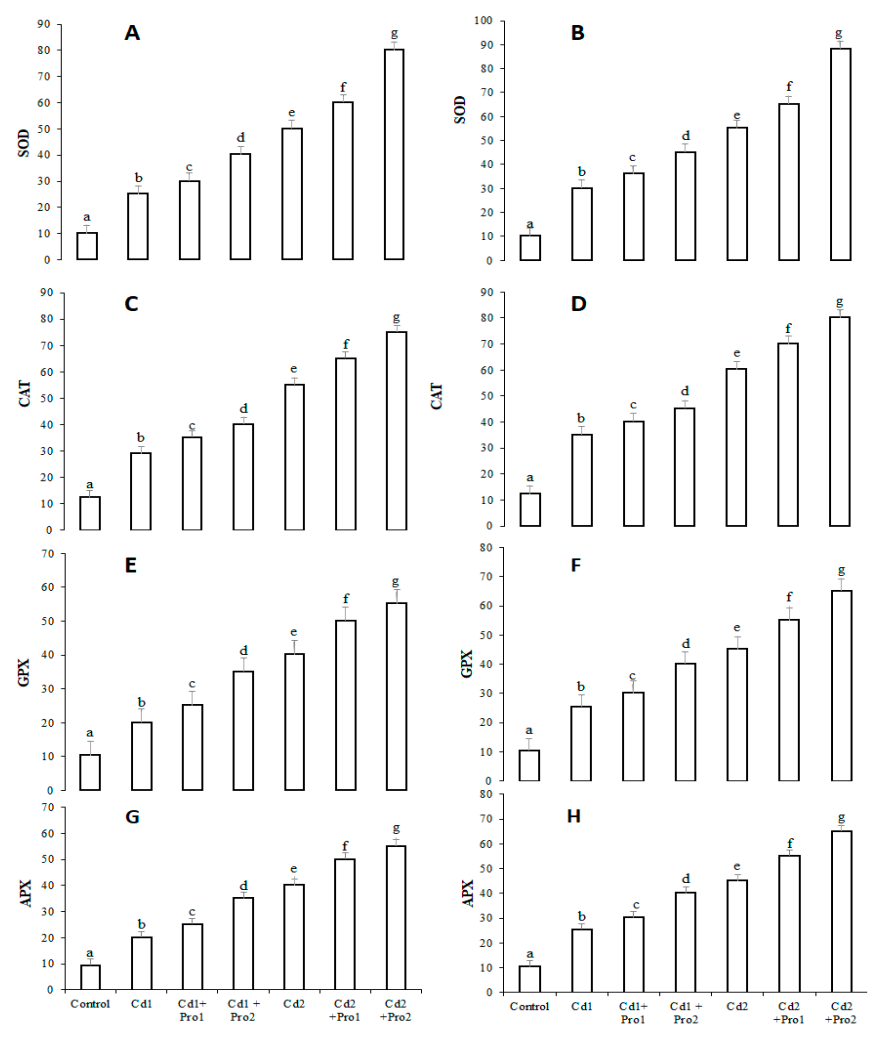
Superoxide dismutase (SOD) activity (U mg^−1^ protein), peroxide reductase catalase (CAT) activity (U mg^−1^ protein), glutathione peroxidase (GPX) activity (U mg^−1^ protein), and ascorbate peroxidase (APX) activity (U mg^−1^ protein) in leaves (**A**,**C**,**E**,**G**) and roots (**B**,**D**,**F**,**H**) of pigeon pea plants subjected to various cadmium and exogenous proline treatments. Bars represent the mean of three replicates ± SD. Cd 1 (4 mg), Cd 2 (8 mg), Cd 1 + Pro 1 (4 mg Cd + 3 mM proline), Cd 1 + Pro2 (4 mg Cd + 6 mM proline), Cd 2 + Pro1 (8 mg Cd + 3 mM proline), Cd 2 + Pro 2 (8 mg Cd + 6 mM proline). Different small letters show significant differences (*p* ≤ 0.05) according to Duncan’s multiple range test.

The antioxidant enzyme activities in the presence of cadmium treatment, alone and combined with exogenous proline, are illustrated in Figure 3. Activities of superoxide dismutase (SOD), peroxide reductase catalase (CAT), ascorbate peroxidase (APX), and glutathione peroxidase (GPX) were significantly higher in cadmium-treated plants than in control plants (*p* ≤ 0.05; Figure 3). These antioxidant enzymes further increased under the combined treatment of exogenous proline and cadmium (Figure 3). The 6 mM exogenous proline in 8 mg/mL Cd irrigation solution treatment increased SOD, CAT, GPX, and APX activities in roots by 35%, 23%, 15%, and 21% and in leaves by 30%, 20%, 15%, and 15.21%, respectively, in comparison with plants treated with Cd 2 alone.

## 3. Discussion

Heavy metals inhibit plants’ growth and decrease their biomass. The inhibition of growth traits among plants is dependent on the nature of the metal, its availability in the medium, the plant tissue, and species [1,2,4]. Cadmium contamination in agricultural land occurs globally, affecting soil nutrients and productivity, and causing health problems in consumers [3,4]. In our study, different cadmium treatments adversely affected pigeon pea height, leaf area, and dry mass (Table 3). These growth-inhibiting effects of Cd on pigeon pea may be attributed to the significant decrease in photosynthetic activities, nutrient deficiency, and excessive ROS production (Table 1 and Table 2; Figure 1). Previous studies have reported similar reduced growth attributes in other plant species due to cadmium stress [14,15,52,53]. Photosynthesis is plants’ life-sustaining process and is often sensitive to metal stresses. The inhibitory effects of cadmium on photosynthesis have been previously reported [23,54]. Its presence in leaves influences the transpiration rate, carbon fixation, and stomatal conductance.

Cadmium reduces stomatal density, decreases stomatal pore size, and affects its normal opening and closing mechanism [21,55,56]. Cadmium ions disturb the thylakoid membrane chain and thus decrease rubisco efficiency in carbon fixation [24,55]. Moreover, the production of elevated H_2_O_2_ contents in the leaves in Cd stress environments may also affect photosynthetic chain performance [55,56]. Chlorophylls play a vital role in the light reaction of photosynthesis. Any environmental factor that affects its performance would lead to a reduction in the photosynthetic process. Cadmium is involved in chlorophyll reduction by inhibiting its biosynthetic enzymes (i.e., protochlorophyllide reductase and δ-aminolaevulinic acid dehydratase) [57]. Cadmium substitutes the central Mg^2+^ molecule from chlorophyll due to its binding nature. The Mg^2+^ atom substitution from chlorophyll reduces its absorption capacity [58]. In our current experiment, photosynthetic attributes and pigments were reduced in pigeon pea under different cadmium treatments (Table 2). Our results are consistent with previous findings, where similar reduced photosynthetic attributes have been observed in other plant species [24,55,56,59].

Macronutrients are required for the normal growth and biochemical processes of plants. Heavy metals, at elevated concentrations, inhibit the uptake and transport of essential nutrients [18,19,20]. Cadmium influences membrane permeability and decreases H^+^-ATPase activity, leading to a reduction in nutrient uptake [60]. Its ions compete with other essential nutrients in apoplast and root vacuoles, affecting nutrient transport and distribution among the plant organs [18,19]. Pigeon pea exposure to cadmium treatments reduced the uptake of Mg^2+^, K^+^, and Ca^2+^ (Table 1). This reduction in essential nutrients evidenced the toxicity of cadmium and its interruption of essential micronutrient uptake and distribution.

The uptake, accumulation, and distribution of metals among plant tissues depend on climatic conditions, stress level, exposure time, and species [61]. From a species perspective, hyperaccumulators are known for their ability to transport and maintain a high amount of heavy metals in their upper tissues [62]. Pigeon pea plants’ exposure to cadmium treatments retained higher Cd amounts in their root tissues (Table 1). Various factors may be involved in this higher content of cadmium in pigeon pea roots, including the negatively charged surface of the cell wall, chelating in the cytosol or compartmentalization in vacuoles, cross-linkage of cadmium with the carboxyl group of the cell wall protein, and the interaction of cadmium with soluble and non-soluble protein thiol groups [63,64].

Metal stress in plants produces excessive amounts of reactive oxygen species (ROS), which lead to oxidative stresses [28,29]. The consequences of oxidative stress involve lipoperoxidation and oxidative damage to membrane lipids, enzymes, chloroplasts, and nucleic acids [28,29]. ROS molecules are extremely reactive, unstable, and toxic at elevated levels. Hydrogen peroxide originates from the dismutation of superoxide (O_2_^−^), the precursor of other ROS. It has a specific characteristic compared with other ROS molecules: it is uncharged and non-radical. These features determine its stability under various physiological conditions. Hydrogen peroxide acts either as a signaling molecule or as an oxidative damage inducer at the cellular level on the basis of its production rate [65]. Cadmium stress-induced oxidative stress in pigeon pea was measured by the elevated contents of hydrogen peroxide, thiobarbituric acid reactive substances, and electrolyte leakage (Figure 2). Our findings are consistent with previous studies, where similar oxidative damage under cadmium exposure in other plant species has been reported [14,15,16].

Endogenous proline content was significantly higher in pigeon pea under different cadmium treatments (Figure 2). Proline accumulates in relative amounts under stress conditions. Proline accumulation is an adaptive strategy to counter oxidative stress injuries through scavenging free radicals, maintaining osmotic balance, sustaining photosystem II, and regulating cellular redox potential [38,39,40]. The possible reasons for increased proline content might be a boost in glutamate synthesis and slowed protein oxidation rate [66,67]. The observed increase in proline content in pigeon pea agrees with the previous findings of Singh et al. [68].

Cadmium stress significantly increased superoxide dismutase, peroxide reductase, catalase ascorbate peroxidase, and glutathione peroxidase activities in pigeon pea (Figure 3). Antioxidant enzymes play an important role in plant defense to stress conditions. These are specific enzymes that serve as ROS scavengers in sub-cellular compartments [32,33,34]. Superoxide dismutase (SOD) is considered to be the first line of defense against ROS-induced stresses, where it is involved in the conversion of superoxide radicals (O_2_^−^) into H_2_O_2_. Superoxide radicals are the precursors of the other ROS [69]. Catalase (CAT) and glutathione peroxidase (GPX) also contribute to H_2_O_2_ scavenging during oxidative stress. Ascorbate peroxidase (APX) reduces H_2_O_2_ to H_2_O by using ascorbate as the electron donor and produces dehydroascorbate. The latter is converted back to ascorbate through reduced GSH as a vital electron donor [70]. This boost in antioxidant enzymes in pigeon pea reflects the enzymatic mechanisms in response to cadmium-induced oxidative stresses. Previous studies have reported similar increases in antioxidant enzymes under Cd stress in other plant species [15,24,71].

Proline is a multifunctional organic molecule that participates in several physiological and biochemical processes, including stress tolerance, ROS scavenging, and signaling [39,40,41]. Exogenous proline in the cadmium irrigation solution significantly reduced cadmium contents in plant tissues (Table 1). The possible cadmium reduction mechanism in pigeon pea might be the formation of a barrier that restricted cadmium influx in plant tissues [52]. According to Sharma et al., Cd ion entrance was reduced due to metal–proline complex formation in their in vitro study on alleviation [72]. In addition, Islam et al. [73] and Chen et al. [74] reported similar reduced cadmium and copper contents in tobacco and rice seedlings under in vitro conditions.

Pigeon pea plants treated with exogenous proline in Cd irrigation solution displayed improvement in terms of growth traits (Table 2). These effects of proline on plant growth may be correlated with enhanced nutrient uptake (Mg^2+^, K^+^, and Ca^2+^), elevated photosynthetic attributes, and decreased oxidative stresses (Table 1 and Table 2; Figure 1). According to previous findings, exogenous proline in stress environments can enhance plasma membrane H^+^-ATPase activity, which plays a crucial role in nutrient absorption [71]. Its application in a metal stress medium led to proline complex formation, reducing metal entry into plant tissues, thus creating more space for the free movement of nutrients [72]. Several studies have highlighted the ameliorative effects of exogenous proline on photosynthetic attributes under stress conditions [42,44,45,46,47,48,75]. Exogenous proline in stress environments can stabilize the mitochondrial transport chain, enhance rubisco activity in carbon fixation, and thus accelerate the photosynthesis dark reaction [75]. Furthermore, it can restore stomatal opening by flagging the abscisic acid-binding ability to specific proteins in membrane guard cells [76]. It also participates in increasing K^+^ in guard cells, which are important for stomatal opening, as previously reported [77]. Exogenous proline increased superoxide dismutase, peroxide reductase, catalase ascorbate peroxidase, glutathione peroxidase, and endogenous proline contents in pigeon peas under cadmium stress and decreased cadmium-induced oxidative damage [78]. Moreover, a balance was observed in antioxidant enzyme activity and oxidative stress indicators in exogenous proline-supplemented pigeon pea plants. The stimulatory effects of exogenous proline on induced systemic tolerance (IST) in pigeon pea cannot be ignored. Induced systemic tolerance (IST) is another possible mechanism through which the damage caused by adverse Cd conditions in pigeon pea can be reduced, as it increases the abiotic stress tolerance capacity of plants [79].

## 4. Materials and Methods

### 4.1. Chemicals and Reagents

Acetone standard grade (AR), ethanol, sulfosalicylic acid, ortho-phosphoric acid, ascorbic acid, and glacial acetic acid were purchased from Xilong Scientific Chemicals (XSC; Shanghai, China); trichloro acetic acid (TCA) was obtained from Thermo Fisher Scientific China (Shanghai chemical); thiobarbituric acid (TBA) was obtained from Sinopharm Chemical reagents; potassium phosphate (K_3_PO_4_) and potassium iodide (KI) were purchased from Sichuan chemical Reagent (Sichuan, China); toluene was obtained from Shanghai Chemex chemicals; EDTA and riboflavin were obtained from Shanghai Huayi Bio-Lab; nitro blue tetrazolium (NBT) was provided by a lab supplier (Shanghai); GR was obtained from glutathione manufacturers and suppliers; and NADPH, proline, FeSO_4_, and HCL were purchased from Sinopharm Chemical Reagent (Shanghai, China). All reagents and chemicals were of analytical grade.

### 4.2. Seed Collection, Sterilization, Germination, and Experimental Site

The experiment was conducted at the Ecology Department of Northeast Forestry University, Harbin, P.R. China. Pigeon pea seeds (hybrid) were obtained from the Traditional Chinese Medicinal University, Harbin. The obtained seeds were initially sterilized with 80% ethanol for 30 s and shifted to 5% NaOCl solution for 10 min and then washed three times with distilled water. The sterilized seeds were germinated in small pots filled with moist soil and used in the experiment after germination.

### 4.3. Soil Collection, Characterization, and Pot Preparation

The soil was collected from the backyard of the Northeast Forestry University. The collected soil was dried for one week, ground properly using a mortar, and passed through a 2 mm sieve tube. Soil characterization was performed using the procedure of Hunter et al. [80]. Its characterization was as follows: sand, 73.8%; clay, 12.2%; silt, 11.4%; soil pH, 7.1; electrical conductivity, 2.8 mScm^−1^; organic matter, 13.52 g kg^−1^; available phosphorus, 64.63 mg kg^−1^; available potassium, 79.39 mg kg^−1^; total nitrogen, 75.62 mg kg^−1^; and soil Cd, 0.10 mg kg^−1^. We filled 7 kg of the physiochemically characterized soil in polythene pots (55 × 30 cm).

### 4.4. Experimental Design, Treatment Procedure, and Seedling Shifting

The germinated uniform five-week-old seedlings were vigilantly shifted into 32 pots (one per pot) filled with 7 kg of physiochemically characterized soil. The experiment was designed in a randomized complete block design (RBDC) with three replicates. The greenhouse growth conditions were: 28/21 °C temperature (day/night), relative humidity of 65–75%, and average daily photosynthetic active radiation of 410–570 m^−2^s^−1^. The seedling pots were divided into seven groups for the different cadmium and exogenous proline treatments. Cd and exogenous proline were applied to the seedlings for eight weeks (from August start to October end) in the following pattern:

1. Group a: Plant was kept as control (without Cd and exogenous proline treatment) and irrigated with distilled water when required.
2. Group b: Plants were irrigated with 200 mL distilled water containing 4 mg CdCl_2_ (Cd1) once per week.
3. Group c: Plants were irrigated with 200 mL distilled water containing 8 mg CdCl_2_ (Cd 2) once per week.
4. Group d: Plants were irrigated with 200 mL distilled water containing 4 mg CdCl_2_ + 3 mM proline (Cd 1 + Pro 1) once per week.
5. Group e: Plants were irrigated with 200 mL distilled water containing 4 mg CdCl_2_ + 6 mM proline (Cd 1 + Pro 2) once per week.
6. Group f: Plants were irrigated with 200 mL distilled water containing 8 mg CdCl_2_ + 3 mM proline (Cd 2 + Pro 1) once per week.
7. Group g: Plants were irrigated with 200 mL distilled water containing 8 mg CdCl_2_ + 6 mM proline (Cd 2 + Pro 2) once per week.

The plants were irrigated with distilled water according to the requirements. The treatment procedure was terminated after eight weeks.

Group b (Cd 1) received 32 mg Cd, Group c (Cd 2) received 64 mg Cd, Group d (Cd 1 + Pro 1) received 32 mg Cd and 24 mM exogenous proline, Group e (Cd 1 + Pro 2) received 32 mg Cd and 48 mM exogenous proline, Group f (Cd 2 + Pro 1) received 64 mg Cd and 24 mM exogenous proline, and Group g (Cd 2 + Pro 2) received 64 mg Cd and 48 mM exogenous proline. The Northeast Forestry University Ecology Department provided the distilled water used in the treatment and irrigation process with the following composition: EC = 1.2 dsm^−1^, pH = 7.1, Na^+^ = 139 mg L^−1^, K^+^ = 243 mg L^−1^, Cl^−^ = 219 mg L^−1^, and Mg^+2^ = 54 mg L^−1^.

### 4.5. Determination of Gaseous Exchange

Measurement of gaseous exchange, that is, net photosynthesis (Pn), stomatal conductance (Gs), and transpiration rate (E), was conducted on a clear day at 27 °C and 65–71% relative air moisture during the daytime (from 10:50 a.m. to 12:50 p.m.) on expanded leaves using a portable gas exchange system (Li-Cor model 6200, Lincoln, Dearborn, MI, USA).

### 4.6. Chlorophylls Determination

We ground 300 mg of fresh leaves in 80% acetone (15 mL) using a pestle and mortar and homogenized them for 1 min at 1000 rpm. After homogenization, the samples were filtered, and the filtrate was centrifuged for 10 min at 2500 rpm at 4 °C. The filtrate was taken, and absorbance was checked at 663 and 645 nm for chlorophyll a and b, respectively, whereas acetone 80% was used in the case of a blanket. Total chlorophyll a and b were calculated using the Lichtentaler formula [81].

### 4.7. Measurement of Oxidative Stress Indicators: Lipid Peroxidation (TBRS), Hydrogen Peroxide (H_2_O_2_), and Electrolyte Leakage (EL)

#### 4.7.1. Lipid Peroxidation (TBRS)

Lipid peroxidation (TBRS) contents were measured following the method of Delmail et al. [82]. Fresh samples of 0.5 g, including roots and leaves separately, were taken, ground, and homogenized separately in 10 mL of 0.1% trichloroacetic acid (TCA) and centrifuged at 12,000 rpm for 5 min. To an aliquot (1.0 mL) of the supernatant, 4 mL of 0.5% thiobarbituric acid (TBA) in 20% TCA was added. The blend was heated at 95 °C for 30 min and immediately cooled in an ice bath. Centrifugation was carried out at 12,000 rpm for 10 min and absorbance was measured at 532 nm by a UV spectrophotometer. The non-specific value at 600 nm absorption was subtracted. The total MDA contents were calculated by the extinction coefficient at 155 mM^−1^cm^−1^ and are expressed as nmol MDA per gram of fresh weight.

#### 4.7.2. Hydrogen Peroxide (H_2_O_2_)

Hydrogen peroxide (H_2_O_2_) contents were measured following the procedure of Junglee et al. [83]. Fresh samples (roots and leaves) of 1 g were ground in 2.0 mL of 0.1% trichloroacetic acid (TCA) solution (*w*/*v*) in an ice bath. The samples were then homogenized and centrifuged at 4 °C for 15 min at 12,000 rpm. Consequently, 0.30 mL of the supernatant was taken, and 0.85 mL of buffer solution containing potassium phosphate (pH 7.0) of 10 Mm and 1 M potassium iodide (1 mL) was added. The total volume was finalized to 2.1 mL in each tube. Absorbance was measured using a UV–Vis spectrophotometer at 390 nm, and the H_2_O_2_ content was calculated from its standard curve. Hydrogen peroxide (H_2_O_2_) contents are expressed as µmol of H_2_O_2_ g^−1^ fresh weight (FW).

#### 4.7.3. Electrolyte Leakage (EL)

For the determination of electrolyte leakage (EL), the procedure of Lutts et al. [84] was followed. Fresh samples of leaves and roots were taken separately and sliced into tiny fragments, up to 5 mm. These 5 mm pieces were placed in a test tube filled with 10 mL deionized water and incubated for 24 h on a rotary shaker at room temperature. Subsequently, the preliminary EC1 was measured. Again, the samples were kept in an oven for 120 min at 90 °C, collected, and cooled at 25 °C. After cooling, the second EC2 was assessed. Total EL was estimated using the following formula:

EL (%) = (EC1/EC2) × 100.

### 4.8. Proline Determination

Proline content was estimated according to the method of Bates et al. [85]. Fresh leaves (250 mg) and roots (300 mg) were homogenized separately in 10 mL of sulfosalicylic acid (3% *w*/*v*), and the homogenate was centrifuged for 10 min at 3000 rpm. To 2 mL of the supernatant, 2 mL of 6 M ortho-phosphoric acid, 2 mL of acid ninhydrin, and 2 mL of glacial acetic acid were added. The mixture was kept in a water bath at 100 °C for 1 h and transferred into a separating funnel. Then, 4 mL of toluene was added, the mixture was shaken vigorously, and a pink layer appeared. Absorbance was measured at 520 nm by a UV spectrophotometer. The proline concentrations were estimated from a standard curve and are expressed as µmol g^−1^ fresh mass.

### 4.9. Antioxidant Enzymes Extraction

Antioxidant enzymes were quantified using fresh root and leaf samples of the plants by UV–Vis spectrophotometry. Plant samples of 0.5 g were soaked in liquid nitrogen and homogenized in 2 mL of extraction buffer containing 100 mM potassium phosphate (7.4 PH), 0.1 mM EDTA, and 10 mM ascorbic acid. The blend was centrifuged at 4 °C for 15 min at 15,000 rpm. The supernatant was gathered and used for the estimation of antioxidant enzyme activities.

### 4.10. Enzymes Quantifications

The SOD activity (EC 1.15.1.1) was assessed by the Reis method [86]. The reaction blend consisted of 0.17% enzyme extracts, 50 mM potassium phosphate (pH 7.8), 10 mM methionine, 33 mM NBT, 3.3 mM riboflavin, and 0.66 mM EDTA in a 3 mL final volume. After incubating the reaction mixture at 28 °C for half an hour under fluorescent light, the absorbance was measured at 560 nm.

CAT activity (EC 1.11.1.6) was monitored following the Aebi method [87] by observing a decrease in absorbance for 60 s at 240 nm. The reaction blend contained 0.35% enzyme extract, 20 mM H_2_O_2_, and 50 Mm potassium phosphate buffer (pH 7.8) in a 3 mL final volume. For the reaction’s initiation, the enzymatic extract was added, and CAT activity was determined from the extinction coefficient (i.e., 39.4 mM^−1^ cm^−1^)_._

GPX activity (GPX, E.C. 1.11.1.9) was determined using the method of Hossain et al. [88]. The decrease in absorbance at 340 nm for 60 s was monitored using a UV–Vis spectrometer. The reaction blend contained 0.63% of enzyme extract, 50 mM sodium phosphate (pH 7), 0.1 mM EDTA, 0.1 mM FeSO_4_, 0.1 mM NADPH, 0.1 mM GSH, 0.1 unit of GR, and 0.1 mM H_2_O_2_ in a 3 mL final volume. The reaction was commenced by adding enzymatic extract, and GPX was determined by the extinction coefficient (i.e., 6.62 mM^−1^cm^−1^).

APX activity (APX, E.C. 1.11.1.11) was calculated following Mizuno et al. [89] by observing a decrease in absorbance for 60 s at 290 nm. The reaction solution contained 50 mM potassium phosphate buffer, 20 mM sodium ascorbate, 20 mM H_2_O_2_, 0.1 mM EDTA, and 0.41% enzymatic extract in a 3 mL final volume. For the initiation of the reaction, H_2_O_2_ was added, and APX activity was determined by the extinction coefficient (i.e., 2.81 mM^−1^cm^−1^).

### 4.11. Measurement of Growth Parameters

Plant growth parameters (i.e., height, leaf and root dry mass, and leaf perimeter) were measured after harvesting. Plant height was measured using a meter rod. Leaf perimeter was measured by a leaf area meter (LI-2000, LI-COR, Lincion, NE, 68504, USA). Leaves and roots were oven dried until reaching a constant weight, after which their mass was recorded.

### 4.12. Elemental Analysis

Elemental analysis of the plant tissues (i.e., leaves and roots) was performed as described by Bankaji et al. [90]. In brief, 500 mg of dry root and leaf samples was separately oven dried for 3 h at 250 °C and digested in 1 M HNO_3_ (10 mL). The obtained solution was attuned by 25 mL distilled water and filtered. The samples were analyzed using an atomic absorption spectrophotometer (Perkin Elmer A Analyst 300, USA) to detect Cd, K, Ca, and Mg in the digested extracts of the leaf and root samples.

### 4.13. Statistical Analysis

Data were recorded from three replicates and analyzed by one-way analysis of variance (ANOVA) using the SPSS v. 21.0 software package. Mean separations were executed by Duncan’s multiple range tests. Significant differences were considered significant at *p* ≤ 0.05.

## 5. Conclusions

Cadmium stress is a severe environmental problem that affects plant physiology and biochemical processes and reduces productivity in the agricultural sector. Exogenous proline illustrated its important role in conferring tolerance and adaptation to pigeon pea plants under Cd stress in this study. It exhibited potential for addressing crop security-related issues, as exogenous proline against cadmium stress reduced its accumulation, enhanced the photosynthetic apparatus, and triggered antioxidant defense mechanisms, which play active roles in ensuring pigeon pea plant survival under cadmium stress. The development of heavy metal-tolerant species through genetic engineering is an essential but time-consuming procedure, whereas exogenous proline supplementation for alleviating metal stresses in plants presents a novel opportunity. Hence, its application may be recommended to mitigate stress in other plant species grown on metal-contaminated soil.

## Figures and Tables

**Table 1:** Impacts of different cadmium and exogenous proline treatments on cadmium accumulation (μg g^−1^ dry weight (DW)) and nutrient uptake (K^+^ (mg g^−1^ DW), Ca^2+^ (mg g^−1^ DW), and Mg^2+^ (mg g^−1^ DW)) in the leaves and roots of pigeon pea.

Treatment	Leaves				Roots			
	Cd^2+^	K^+^	Ca^2+^	Mg^2+^	Cd^2+^	K^+^	Ca^2+^	Mg^2+^
Control	0.09 ± 0.15 ^a^	16.59 ± 0.93 ^c^	11.14 ± 1.03 ^c^	4.41 ± 1.11 ^d^	0.26 ± 0.07 ^a^	18.15 ± 0.15 ^c^	9.01 ± 1.07 ^g^	3.22 ± 0.11 ^a^
Cd 1	76.24 ± 1.11 ^e^	16.59 ± 0.95 ^c^	8.04 ± 0.90 ^b^	5.04 ± 0.14 ^e^	155.21 ± 1.97 ^d^	18.19 ± 0.11 ^d^	6.82 ± 0.8 ^c^	3.32 ± 0.09 ^f^
Cd 1 + Pro 1	51.47 ± 0.17 ^c^	17.03 ± 1.06 ^e^	8.96 ± 1.04 ^d^	5.51 ± 0.161 ^f^	108.6 ± 2.12 ^c^	18.27 ± 0.18 ^f^	7.47 ± 0.21 d	3.46 ± 0.09 ^d^
Cd 1 + Pro 2	27.55 ± 0.83 ^b^	17.17 ± 0.99 ^f^	10.36 ± 0.93 ^e^	5.74 ± 0.73 ^f^	56.06 ± 0.15 ^b^	18.34 ± 0.15 ^e^	8.65 ± 0.8 f	3.85 ± 1.01 ^e^
Cd 2	161.26 ± 1.70 ^g^	13.28 ± 1.08 ^a^	6.84 ± 1.07 ^a^	2.45 ± 0.14 ^a^	329.3 ± 3.85 ^f^	15.15 ± 0.26 ^a^	5.36 ± 0.650 a	2.07 ± 0.11 ^g^
Cd 2 + Pro 1	103.47 ± 1.12 ^f^	14.65 ± 0.93 ^b^	7.97 ± 0.24 ^f^	3.17 ± 1.15 ^b^	267.14 ± 2.04 ^e^	16.31 ± 0.1 ^b^	6.78 ± 0.106 ^b^	2.52 ± 0.07 ^b^
Cd 2 + Pro 2	52.39 ± 0.99 ^d^	15.96 ± 1.04 ^d^	9.64 ± 1.03 ^g^	3.95 ± 2.18 ^c^	141.84 ± 1.53 ^g^	18.10 ± 0.72 ^c^	8.43 ± 0.85 ^e^	3.11 ± 0.81 ^c^

Values represent the means of three replicates ± SD. Cd 1 (4 mg), Cd 2 (8 mg), Cd 1 + Pro 1 (4 mg Cd + 3 mM proline), Cd 1 + Pro 2 (4 mg Cd + 6 mM proline), Cd 2 + Pro 1 (8 mg Cd + 3 mM proline), Cd 2 + Pro 2 (8 mg Cd + 6 mM proline). Different small letters in columns show significant differences and the same small letters show non-significant differences (*p* ≤ 0.05) according to Duncan’s multiple range test.

**Table 2:** Different cadmium and exogenous proline effects on net photosynthesis (µmol of CO_2_ m^−2^ s^−1^), stomatal conductance (mmol of H_2_O_2_ m^−2^ s^−1^), transpiration rate (mmol of H_2_O_2_ m^−2^ s^−1^), chlorophyll a (mg g^−1^ fresh weight (FW)), and chlorophyll b (mg g^−1^ FW) in pigeon pea.

Treatment	Net Photosynthesis	Stomatal Conductance	Transpiration Rate	Chlorophyll a	Chlorophyll b
Control	21.41 ± 0.11 ^g^	150.21 ± 0.14 ^d^	7.65 ± 0.09 ^g^	2.49 ± 0.06 ^a^	1.10 ± 0.09 ^b^
Cd1	16.72 ± 1.04 ^d^	121.15 ± 1.01 ^b^	5.26 ± 0.16 ^c^	1.78 ± 0.09 ^f^	0.87 ± 0.08 ^a^
Cd1 + Pro1	18.91 ± 0.09 ^e^	128.31 ± 0.09 ^c^	5.94 ± 1.10 ^d^	1.90 ± 1.03 ^d^	0.98 ± 0.11 ^a^
Cd1 + Pro2	20.65 ± 2.10 ^f^	137.09 ± 0. 21 ^g^	6.32 ± 0.18 ^f^	2.18 ± 0.17 ^e^	1.03 ± 0.99 ^c^
Cd2	13.76 ± 0.18 ^a^	105.24 ± 1.42 ^a^	3.92 ± 0.06 ^a^	1.10 ± 0.12 ^b^	0.50 ± 1.01 ^f^
Cd2 + Pro1	15.95 ± 0.09 ^b^	113.45 ± 0.09 ^f^	4.70 ± 0.09 ^b^	1.49 ± 0.09 ^c^	0.72 ± 1.07 ^g^
Cd2 + Pro2	18.90 ± 0.15 ^e^	121.16 ± 1.32 ^b^	5.95 ± 1.11 ^d^	1.91 ± 1.11 ^d^	0.97 ± 0.09 ^d^

Values represent the means of three replicates ± SD. Cd 1 (4 mg), Cd 2 (8 mg), Cd 1 + Pro 1 (4 mg Cd + 3 mM proline), Cd 1 + Pro 2 (4 mg Cd + 6 mM proline), Cd 2 + Pro 1 (8 mg Cd + 3 mM proline), Cd 2 + Pro 2 (8 mg Cd + 6 mM proline). Different small letters in columns show significant differences and the same small letters show non-significant difference (*p* ≤ 0.05) according to Duncan’s multiple range test.

**Table 3:** Plant height (cm), leaf perimeter (mm), and leaf and root dry weight (g) of pigeon pea subjected to different cadmium and exogenous proline treatments.

Treatment	Plant Height	Leaf Perimeter	Leaves Dry Mass	Roots Dry Mass
Control	61.35 ± 0.07 ^g^	155.29 ± 0.06 ^f^	7.41 ± 0.09 ^f^	9.80 ± 0.09 ^a^
Cd 1	55.05 ± 1.95 ^d^	125.46 ± 0.15 ^b^	5.02 ± 0.18 ^c^	7.65 ± 1.21 ^b^
Cd 1 + Pro 1	57.86 ± 0.14 ^e^	136.28 ± 2.17 ^d^	5.88 ± 0.09 ^d^	8.05 ± 0.07 ^d^
Cd 1 + Pro 2	59.70 ± 2.15 ^f^	147.25 ± 1.12 ^e^	6.47 ± 1.18 ^e^	8.96 ± 0.13 ^c^
Cd 2	54.95 ± 0.17 ^d^	119.19 ± 0.19 ^a^	4.32 ± 2.01 ^a^	6.27 ± 0.10 ^d^
Cd 2 + Pro 1	56.41 ± 1.66 ^b^	127.32 ± 0.24 ^c^	4.99 ± 0.19 ^b^	6.89 ± 0.10 ^b^
Cd 2 + Pro 2	58.86 ± 0.21 ^c^	139.45 ± 0.13 ^g^	5.88 ± 1.16 ^d^	7.66 ± 1.92 ^b^

Values represent the means of three replicates ± SD. Cd 1 (4 mg), Cd 2 (8 mg), Cd 1 + Pro 1 (4 mg Cd + 3 mM proline), Cd 1 + Pro 2 (4 mg Cd + 6 mM proline), Cd 2 + pro 1 (8 mg Cd + 3 mM proline), Cd 2 + Pro 2 (8 mg Cd + 6 mM proline). Different small letters in columns show significant differences and same small letters show non-significant differences (*p* ≤ 0.05) according to Duncan’s multiple range test.
